# Evaluation of patients with painful total hip arthroplasty using combined single photon emission tomography and conventional computerized tomography (SPECT/CT) – a comparison of semi-quantitative versus 3D volumetric quantitative measurements

**DOI:** 10.1186/s12880-017-0204-x

**Published:** 2017-05-08

**Authors:** Emilienne Barthassat, Faik Afifi, Praveen Konala, Helmut Rasch, Michael T. Hirschmann

**Affiliations:** 1 Department of Orthopaedic Surgery and Traumatology, Kantonsspital Baselland Bruderholz, Liestal, Laufen Switzerland; 2 Fellow- Musculoskeletal Radiology, The Robert Jones and Agnes Hunt Orthopaedic Hospital NHS Foundation Trust, Oswestry, UK; 3 Institute for Radiology and Nuclear Medicine, Kantonsspital Baselland-Bruderholz, Bruderholz, Switzerland; 4 Basel University, Basel, Switzerland

**Keywords:** Hip, SPECT/CT, Total hip arthroplasty, Total hip replacement, Pain, Localization scheme, Bone tracer uptake intensity, Quantification, Three-dimensional

## Abstract

**Background:**

It was the primary purpose of our study to evaluate the inter- and intra-observer reliability of a standardized SPECT/CT algorithm for evaluating patients with painful primary total hip arthroplasty (THA). The secondary purpose was a comparison of semi-quantitative and 3D volumetric quantification method for assessment of bone tracer uptake (BTU) in those patients.

**Methods:**

A novel SPECT/CT localization scheme consisting of 14 femoral and 4 acetabular regions on standardized axial and coronal slices was introduced and evaluated in terms of inter- and intra-observer reliability in 37 consecutive patients with hip pain after THA. BTU for each anatomical region was assessed semi-quantitatively using a color-coded Likert type scale (0-10) and volumetrically quantified using a validated software. Two observers interpreted the SPECT/CT findings in all patients two times with six weeks interval between interpretations in random order. Semi-quantitative and quantitative measurements were compared in terms of reliability. In addition, the values were correlated using Pearson`s correlation. A factorial cluster analysis of BTU was performed to identify clinically relevant regions, which should be grouped and analysed together.

**Results:**

The localization scheme showed high inter- and intra-observer reliabilities for all femoral and acetabular regions independent of the measurement method used (semiquantitative versus 3D volumetric quantitative measurements). A high to moderate correlation between both measurement methods was shown for the distal femur, the proximal femur and the acetabular cup. The factorial cluster analysis showed that the anatomical regions might be summarized into three distinct anatomical regions. These were the proximal femur, the distal femur and the acetabular cup region.

**Conclusions:**

The SPECT/CT algorithm for assessment of patients with pain after THA is highly reliable independent from the measurement method used. Three clinically relevant anatomical regions (proximal femoral, distal femoral, acetabular) were identified.

## Background

Combined single photon emission computerized tomography and conventional CT (SPECT/CT) promises the combined assessment of anatomical and functional information and hence its value is increasingly recognized in orthopaedics [1–23].

SPECT/CT has been reported to be beneficial in identifying the cause of patients`pain after total knee arthroplasty, patients with chondral or osteochondral lesions, before and after high tibial osteotomy and after ACL reconstruction [1–23]. Although SPECT/CT has also been used in patients with pain after total hip arthroplasty (THA) there is only scarce evidence about the optimal diagnostic algorithm and method of bone tracer uptake (BTU) analysis [24–27].

Due to its specific characteristics SPECT/CT is more sensitive and specific than SPECT and CT alone. Clearly, it is the accurate anatomical localization of the SPECT-tracer uptake using the CT as reference map that promises improved diagnostic confidence, particularly in patients with pain after joint replacement surgery [17, 20, 23]. Detection of mechanical or septic loosening of THA even in early stages might be facilitated. In addition, it could provide the surgeon with information on the position of THA components.

Recently our group has described and validated a standardized diagnostic algorithm using SPECT/CT in patients with pain after total knee arthroplasty including analysis of bone tracer activity and position and alignment of the TKA [14, 17, 20, 23, 28]. However, no such diagnostic algorithm has been reported or validated in patients with pain after THA.

In addition, in clinical practice it is a pertinent question if quantification of BTU in SPECT/CT offers so much more information than formerly semi-quantitative methods to recommend its daily use. There is no current study comparing semi-quantitative and quantitative measurement of BTU in patients with pain after THA.

Hence, it was the primary purpose of our study to evaluate the inter- and intra-observer reliability of a standardized SPECT/CT algorithm for evaluating patients with painful primary THA. The secondary purpose was a comparison of semi-quantitative and 3D volumetric quantification method for assessment of BTU in those patients.

## Methods

### Patients

A consecutive series of 37 patients (m:f = 16:21, mean age ± standard deviation 71 ± 11 years) presenting with hip pain after primary THA were prospectively collected and retrospectively included in this study. The data from all this patients were collected using our clinical information system (KIS, Erne, Switzerland). All patients had a primary THA with a maximum interval of 6 months from primary THA.

The study was approved by the local ethical committee (EKNZ 205/10). Written informed consent was obtained from all patients.

SPECT/CT was performed using a hybrid system (Symbia T16, Siemens, Erlangen, Germany) with a dual-head gamma camera and an integrated, 16x0.75-mm slice-thickness CT. All patients received a commercial 500–700 MBq Tc-99m-HDP injection (Malinckrodt, Wollerau, Switzerland). Scintigraphic images in anterior-posterior and lateral projection were taken in the perfusion phase (immediately after injection), the soft tissue phase (3–5 min after injection) and the delayed metabolic phase (2–3 h after injection). SPECT/CT was performed with a matrix size of 128x128, an angle step of 32, and a time per frame of 25 s.

The CT protocol was modified according to the Imperial Knee Protocol, which is a low dose CT protocol that includes high-resolution 0.75 mm slices of the knee and 3 mm slices of the hip and ankle joints [29].

**Fig. 1:**
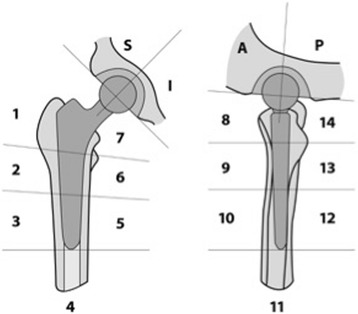
The localization scheme for the Tc-99m-HDP bone tracer activity in patients with painful hips after primary THA

The localization of bone tracer activity was recorded on a standardized localization scheme developed for use in patients after primary THA (Fig. 1). This defines biomechanically relevant regions of the femoral shaft and acetabulum around total hip prosthesis on standardized axial, coronal, and sagittal slices to accurately map areas of increased activity. The anatomical area (femur, acetabulum) is indicated with capital letters (F, A). The femur (F) is divided into fourteen zones with regards to the modified Gruen classification [30].

Each femoral zone is represented with a number (1-14). The acetabulum (A) is divided into four zones (superior anterior, superior posterior, inferior anterior, inferior posterior).

The highest activity grading on SPECT/CT for each area of the localization scheme was recorded semiquantitatively (0–10). In addition, it was noted whether the area of tracer activity extended to the bone prosthesis interface. In that case an additional “c” was added to the tracer activity value.

**Fig. 2:**
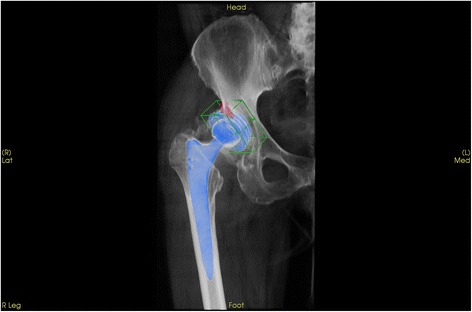
3D volumetric quantification of acetabular cup areas with regards to the localization scheme using the customized software

**Fig. 3:**
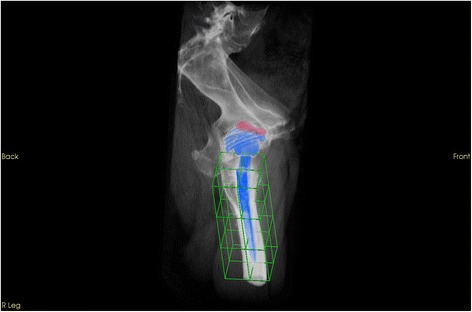
3D volumetric quantification of femoral areas with regards to the localization scheme using the customized software

In addition, BTU was also quantified in 3D using a voxel based measurement method. For BTU analysis (intensity and anatomical distribution pattern) the 3D reconstructed datasets of the delayed SPECT/CT images were used. The anatomical areas represented by a previously validated localization scheme were 3D volumetrically measured in terms of SPECT/CT tracer uptake values (OrthoImagingSolutions Ltd., London, UK) [4, 9]. The tracer activity was quantified in 3D volumetrically as described in Hirschmann et al. (Figs. 1, 2 and 3) [4]. The maximum intensity values were recorded for each anatomical area.

Two observers interpreted the SPECT/CT findings in all patients two times with six weeks interval between interpretations in random order. Both were blinded to results from previous observations. The inter- and intra-observer reliability of the localization scheme and grading of the tracer activity was determined. Semi-quantitative and quantitative measurements were compared in terms of reliability. In addition, the values were correlated using Pearson`s correlation.

Finally, a factorial cluster analysis of BTU was performed to identify clinically relevant regions, which should be grouped and analysed together.

### Statistical analysis

Data were analyzed using SPSS 16.0 (SPSS, Chicago, U.S.A.). Sample size was calculated according to the reported estimates for reliability studies using intraclass correlation coefficients (ICCs) [31].

The median differences in measurements between the two observers (inter-observer) and within the measurements of the first observer (intra-observer) were calculated. The intraclass correlation coefficients for inter- and intra-observer reliability were also calculated. ICC values range from 0 to 1. A value of 1 indicates perfect reliability, 0.81 to 1 very good reliability and 0.61-0.80 good reliability [31]. For all analysis, *p* < 0.05 was considered statistically significant.

## Results

The localization scheme showed high inter- and intra-observer reliabilities for all femoral and acetabular regions independent of the measurement methods (semi-quantitative versus 3D volumetric quantitative measurements). Inter- and intra-observer reliability (intra class correlation- ICC) of 99mTc-HDP-SPECT/CT tracer activity using the localization and semi-quantitative BTU grading scheme for the acetabular and femoral zones (Likert scale 0–10) are presented in Table 1. In mean the femoral regions showed an ICC of 0.981–0.992 for intra-observer reliability and 0.871 for inter-observer-reliability. In mean the acetabular regions showed an ICC of 0.967–0.975 for intra-observer reliability and 0.877 for inter-observer-reliability. The acetabular regions AI and PI showed moderate agreement for intra- (ICC 0.529–0.963) and inter-observer testing (0.401–0.493).

**Table 1:** Inter- and intra-observer reliability (intra class correlation- ICC) of 99mTc-HDP-BTU activity using the localization and semiquantitative BTU grading scheme for the acetabular and femoral zones (Likert scale 0-10)

	Intra-observer-reliability				Inter-observer- reliability	
Location	Observer 1		Observer 2		Observer 1-Observer 2	
	Mean	95% CI	Mean	95% CI	Mean	95% CI
AS	0.954	0.913–0.976	0.975	0.952–0.987	0.900	0.815–0.947
AI	0.963	0.929–0.981	0.529	0.252–0.726	0.401	0.093–0,639
PS	0.967	0.938–0.983	0.949	0.903–0.973	0.950	0.905–0.974
PI	0.878	0.775–0.935	0.790	0.629–0.886	0.493	0.206–0.703
Z1	0.888	0.794–0.941	0.807	0.656–0.895	0.799	0.643–0.891
Z2	0.882	0.783–0.938	0.692	0.478–0.829	0.703	0.495–0.835
Z3	0.781	0.614–0.881	0.929	0.867–0.963	0.866	0.755–0.928
Z4	0.822	0.680–0.904	0.938	0.883–0.968	0.744	0.557–0.860
Z5	0.884	0.786–0.938	0.975	0.951–0.987	0.829	0.693–0.908
Z6	0.877	0.774–0.935	0.792	0.633–0.887	0.733	0.540–0,853
Z7	0.945	0.895–0.971	0.811	0.663–0.898	0.723	0.524–0.847
Z8	0.955	0.914–0.976	0.923	0.855–0.959	0.864	0.752–0.928
Z9	0.937	0.882–0.967	0.833	0.699–0.910	0.601	0.348–0.773
Z10	0.838	0.708–0.913	0.978	0.958–0.989	0.718	0.517–0.844
Z11	0.878	0.777–0.936	0.955	0.914–0.976	0.681	0.461–0.822
Z12	0.881	0.781–0.937	0.964	0.931–0.981	0.846	0.722–0.918
Z13	0.921	0.852–0.958	0.804	0.651–0.894	0.563	0.296–0.748
Z14	0.889	0.796–0.942	0.876	0.772–0.934	0.819	0.675–0.902
Acetabulum	0.975	0.953–0.987	0.967	0.937–0.983	0.877	0.774–0.935
Femur	0.992	0.984–0.996	0.981	0.963–0.990	0.871	0.764–0.932

Inter- and intra-observer reliability (intra class correlation- ICC) of 99mTc-HDP-SPECT/CT tracer activity using the localization and 3D voxel based quantitative BTU grading scheme for the acetabular and femoral zones were for all regions neary perfect (ICCs > 0.90).

The measured values of BTU activity in SPECT/CT for each anatomical region using the semi-quantitative versus 3D volumetric quantitative method are shown in Table 2. The Pearson`s correlation of both BTU measurement methods is presented in Table 3. A high correlation between both measurement methods was found for the distal femur. A moderate correlation was found for the proximal femur and the acetabular cup regions.

**Table 2:** Absolute values (mean, SD, median) of BTU for each anatomical area using 3D volumetric voxel based quantification and semi-quantitative measurement methods

	3D volumetric voxel based quantification of BTU			Semiquantitative measurement of BTU		
Anatomical area	Mean	SD	Median	Mean	SD	Median
AS	860.23	425.19	831.00	2.78	1.80	2.50
AI	817.54	422.63	700.00	1.26	0.89	1.50
PS	822.34	427.53	711.00	2.49	1.70	2.00
PI	871.00	589.04	743.00	1.49	1.06	1.50
Z1	1022.54	626.10	897.00	4.40	2.38	4.00
Z2	820.20	498.31	737.00	3.13	2.02	2.50
Z3	841.40	656.74	690.00	3.23	2.61	2.25
Z4	590.76	471.38	494.00	2.92	2.23	2.50
Z5	851.71	598.32	744.00	3.14	2.48	2.75
Z6	927.97	603.05	750.00	2.83	1.98	2.50
Z7	1125.29	832.16	866.00	3.02	1.95	2.75
Z8	1085.48	779.70	877.00	3.97	2.49	3.50
Z9	899.09	580.63	744.00	3.05	2.10	2.50
Z10	854.37	592.66	753.00	3.12	2.37	2.75
Z11	592.29	454.82	530.00	2.74	2.02	2.50
Z12	848.26	621.53	718.00	3.35	2.72	2.50
Z13	799.03	443.21	740.00	3.08	1.91	2.75
Z14	1043.37	692.13	788.00	3.91	2.25	3.50
Hip	877.53	507.60	774.31	2.01	1.27	2.13
Thigh	842.78	428.19	752.50	3.28	1.96	2.80

**Table 3:** Pearson`s correlations of 99mTc-HDP BTU activity for the acetabular and femoral zones using 3D-quantitative and semi-quantitative methods

	AS Obs	AI Obs	PS Obs	PI Obs	Z1 Obs	Z2 Obs	Z3 Obs	Z4 Obs	Z5 Obs	Z6 Obs	Z7 Obs	Z8 Obs	Z9 Obs	Z10 Obs	Z11 Obs	Z12 Obs	Z13 Obs	Z14 Obs	Hip Obs	Thigh Obs
Voxel AS ratio	0.51	0.29	0.34	0.27	0.19	0.11	0.08	0.08	0.14	0.14	0.17	0.12	0.07	0.17	0.13	0.07	0.17	0.10	0.40	0.14
Voxel Al ratio	0.52	0.29	0.39	0.36	0.12	0.14	0.12	0.16	0.17	0.18	0.17	0.03	0.10	0.19	0.21	0.13	0.22	0.07	0.44	0.16
Voxel PS ratio	0.53	0.29	0.44	0.39	0.19	0.20	0.14	0.22	0.13	0.20	0.24	0.16	0.11	0.17	0.25	0.13	0.30	0.21	0.47	0.21
Voxel PI ratio	0.49	0.42	0.61	0.49	0.01	0.00	0.02	0.07	0.13	0.22	0.18	-0.13	-0.07	0.18	0.11	0.03	0.17	0.08	0.55	0.07
Voxel Z1 ratio	-0.05	-0.01	-0.15	-0.17	0.14	0.26	0.07	0.05	0.09	0.07	0.11	0.10	0.24	0.11	0.09	0.00	0.14	0.09	-0.10	0.12
Voxel Z2 ratio	0.11	0.21	-0.02	-0.01	0.19	0.46	0.37	0.30	0.35	0.29	0.29	0.15	0.41	0.36	0.30	0.32	0.34	0.16	0.07	0.34
Voxel Z3 ratio	0.11	0.28	0.05	0.16	0.25	0.48	0.72	0.63	0.77	0.55	0.46	0.15	0.40	0.77	0.61	0.72	0.49	0.36	0.14	0.60
Voxel Z4 ratio	0.13	0.27	-0.05	0.07	0.16	0.37	0.54	0.62	0.47	0.24	0.22	0.12	0.30	0.42	0.62	0.55	0.22	0.16	0.09	0.40
Voxel Z5 ratio	0.19	0.30	0.12	0.18	0.29	0.50	0.66	0.58	0.77	0.60	0.50	0.19	0.41	0.77	0.58	0.67	0.54	0.43	0.20	0.60
Voxel Z6 ratio	0.13	0.19	0.06	0.04	0.11	0.31	0.37	0.37	0.39	0.41	0.36	0.07	0.30	0.39	0.36	0.33	0.30	0.16	0.11	0.34
Voxel Z7 ratio	-0.03	0.09	-0.06	-0.08	0.05	0.08	0.15	0.15	0.08	0.22	0.25	0.13	0.13	0.14	0.23	0.11	0.13	0.10	-0.03	0.15
Voxel Z8 ratio	-0.07	-0.10	-0.21	-0.19	0.08	0.11	-0.06	0.00	-0.13	-0.06	-0.03	0.14	0.18	-0.14	0.03	-0.11	0.04	-0.02	-0.16	0.00
Voxel z9 ratio	0.25	0.32	0.12	0.19	0.31	0.60	0.51	0.45	0.56	0.51	0.48	0.24	0.61	0.51	0.41	0.49	0.51	0.31	0.23	0.52
Voxel Z10 ratio	0.17	0.30	0.08	0.17	0.21	0.43	0.61	0.57	0.74	0.56	0.46	0.09	0.36	0.73	0.56	0.64	0.48	0.37	0.18	0.55
Voxel Z11 ratio	0.08	0.16	-0.04	0.05	0.14	0.23	0.49	0.57	0.45	0.31	0.25	0.11	0.21	0.40	0.60	0.48	0.24	0.23	0.05	0.38
Voxel Z12 ratio	0.17	0.22	0.04	0.21	0.28	0.48	0.77	0.73	0.77	0.53	0.47	0.20	0.44	0.72	0.70	0.76	0.52	0.38	0.15	0.63
Voxel Z13 ratio	0.14	0.23	0.02	0.03	0.17	0.40	0.37	0.30	0.39	0.33	0.37	0.10	0.36	0.40	0.33	0.32	0.31	0.19	0.10	0.35
Voxel Z14 ratio	0.06	0.12	0.04	0.03	0.19	0.12	0.15	0.18	0.15	0.24	0.34	0.16	0.15	0.17	0.28	0.11	0.19	0.31	0.06	0.22
Voxel Thigh	0.59	0.37	0.51	0.42	0.15	0.13	0.10	0.15	0.16	0.21	0.22	0.05	0.06	0.20	0.20	0.10	0.24	0.13	0.53	0.17
Voxel Hip	0.14	0.26	0.01	0.08	0.25	0.48	0.57	0.55	0.59	0.48	0.45	0.19	0.45	0.58	0.56	0.55	0.45	0.32	0.11	0.52

The factorial cluster analysis showed that the anatomical regions might be summarized into three distinct anatomical regions Table 4. These were the proximal femur, the distal femur and the acetabular cup region.

**Table 4:** Factorial cluster analysis of 3D voxel based quantified BTU values showing three distinct regions (proximal thigh, distal thigh, acetabular cup)

		Absolute BTU				BTU ratio		
		Distal femur	Proximal femur	Acetabular cup		Distal femur	Proximal femur	Acetabular cup
Distal thigh	Z12	0.93	0.18	0.20	Z3 ratio	0.96	0.10	0.01
	Z3	0.93	0.15	0.22	Z5 ratio	0.93	0.14	0.13
	Z5	0.90	0.22	0.23	Z12 ratio	0.93	0.10	0.06
	Z10	0.87	0.22	0.27	Z10 ratio	0.91	0.11	0.17
	Z11	0.85	0.30	0.09	Z4 ratio	0.71	0.26	-0.06
	Z4	0.83	0.22	-0.04	Z11 ratio	0.71	0.29	-0.02
	Z6	0.73	0.38	0.42	Z6 ratio	0.61	0.56	0.20
	Z13	0.81	0.23	0.41	Z13 ratio	0.60	0.55	0.36
	Z9	0.79	0.32	0.38	Z9 ratio	0.56	0.46	0.14
	Z2	0.77	0.28	0.34	Z2 ratio	0.55	0.62	0.13
Proximal thigh	Z8	0.21	0.94	0.11	Z8 ratio	-0.06	0.90	0.08
	Z7	0.31	0.84	0.33	Z1 ratio	0.24	0.82	0.12
	Z14	0.27	0.78	0.39	Z7 ratio	0.21	0.71	0.11
	Z1	0.42	0.76	0.33	Z14 ratio	0.22	0.44	0.22
Acetabular cup	PI	0.06	0.19	0.91	PI ratio	0.02	-0.01	0.91
	PS	0.23	0.20	0.89	AI ratio	0.13	0.16	0.87
	AI	0.36	0.31	0.84	AS ratio	0.12	0.21	0.86
	AS	0.39	0.35	0.71	PS ratio	0.00	0.22	0.79

## Discussion

The most important findings of the present study were twofold. Firstly, a high inter-observer and intra-observer reliability was found for grading and localization of the BTU activity independent of the investigated region. The localization scheme and BTU grading was reliable and easily applicable, which would make it understandable by most clinicians. A reliable localization and grading scheme is needed to standardize the evaluation of SPECT/CT data and make those comparable with each other. The Gruen classification is already widely used for assessment of periprosthetic radiolucencies, hence it was decided to adapt this scheme to the biomechanics of the hip reflecting bone remodeling and integration of the prosthetic hip components. It has also been used by others to report BTU findings in SPECT/CT [26, 32].

In a recent pictorial review by Tam et al. dealing with THA the authors reported their SPECT/CT analysis and reporting system, which is in accordance with the one presented in terms of the localization scheme used [26]. However, there only the two-dimensional localization scheme was used. In this study a modified three-dimensional localization scheme was introduced and has proven highly reliable [26, 33]. The localization scheme showed high inter- and intra-observer reliabilities for both femoral and acetabular regions independent of the measurement methods (semi-quantitative versus 3D volumetric quantitative measurements). Clearly, the 3D volumetric quantification has proven to be as reliable as the standard two-dimensional localization and BTU analysis system.

In a retrospective study Jin et al. investigated the periprosthetic bone remodeling of THA using SPECT/CT [34]. SPECT/CT was reviewed as three-dimensional multiplanar reconstructions with a slice thickness of 4.4 mm [34]. Two-dimensional regions of interest (ROIs) were generated and placed in specific standardized locations for each dataset [34]. All ROI placements and measurements were performed by a single reader to match standardized locations and with the assistance of a ROI template guide [34]. In agreement with the present study they also normalized the absolute measured values by building ratios of the measured value and a value measured at a specific reference regions [34].

Secondly, a high correlation between both measurement methods was found for the distal femur. A moderate correlation was found for the proximal femur and the acetabular cup regions.

The factorial cluster analysis showed that the anatomical regions might be summarized into three distinct anatomical regions These were the proximal femur, the distal femur and the acetabular cup region. In the study by Jin et al. the ROI analysis was done at five different locations (the greater trochanter, the femoral calcar, the mid-stem of the femur, the femoral stem tip and one acetabular region) [34]. The authors choose these locations for analysis as these appeared to be clinically relevant and highly reproducible [34].

However, Jin et al. questioned the need for routine quantification of BTU in patients with THA [34]. Based on their findings it is possible to distinguish between clearly normal and clearly abnormal SPECT/CT images [34]. In difficult cases semi-quantification might be helpful.

In contrast, we believe that a better understanding of bone remodelling after THA reflected by typical BTU pattern distribution will help to improve the reporting and diagnosis when using SPECT/CT. However, until analysis of BTU activity could lead to a better diagnosis we need to achieve a more profound knowledge of normal and abnormal BTU distribution and activity in native and arthroplasty patients.

Another limitation to gain wider acceptance for quantification methods among clinicians is the utility, availability and simplicity of these analysis methods. Clearly, these have to be robust, reliable and easy to perform.

The study bears a few limitations to be considered. This is a well sized small pilot study aiming to evaluate the analysis algorithm in patients with THA undergoing SPECT/CT. The clinical value of the algorithm needs to be further evaluated in larger, homogenous cohorts. The standard deviations are high, which is due to inter-patient variability. A typical findings in metabolic imaging.

## Conclusions

The SPECT/CT algorithm for assessment of patients with pain after THA is highly reliable independent from the measurement method used. Three clinically relevant anatomical regions (proximal femoral, distal femoral, acetabular) were identified.

## Abbreviations

BTU: Bone tracer uptake
ICC: Intraclass coefficient
ROI: Region of interest
SPECT/CT: Single photon emission computerized tomography and conventional computerized tomography
THA: Total hip arthroplasty
